# Biological Contribution to Social Influences on Alcohol Drinking: Evidence from Animal Models

**DOI:** 10.3390/ijerph7020473

**Published:** 2010-02-11

**Authors:** Allison M.J. Anacker, Andrey E. Ryabinin

**Affiliations:** Department of Behavioral Neuroscience, Oregon Health & Science University, 3181 SW Sam Jackson Pk Rd L470, Portland, OR 97239, USA; E-Mail: anackera@ohsu.edu

**Keywords:** ethanol, affiliative behavior, animal model

## Abstract

Social factors have a tremendous influence on instances of heavy drinking and in turn impact public health. However, it is extremely difficult to assess whether this influence is only a cultural phenomenon or has biological underpinnings. Research in non-human primates demonstrates that the way individuals are brought up during early development affects their future predisposition for heavy drinking, and research in rats demonstrates that social isolation, crowding or low social ranking can lead to increased alcohol intake, while social defeat can decrease drinking. Neurotransmitter mechanisms contributing to these effects (*i.e.*, serotonin, GABA, dopamine) have begun to be elucidated. However, these studies do not exclude the possibility that social effects on drinking occur through generalized stress responses to negative social environments. Alcohol intake can also be elevated in positive social situations, for example, in rats following an interaction with an intoxicated peer. Recent studies have also begun to adapt a new rodent species, the prairie vole, to study the role of social environment in alcohol drinking. Prairie voles demonstrate a high degree of social affiliation between individuals, and many of the neurochemical mechanisms involved in regulation of these social behaviors (for example, dopamine, central vasopressin and the corticotropin releasing factor system) are also known to be involved in regulation of alcohol intake. Naltrexone, an opioid receptor antagonist approved as a pharmacotherapy for alcoholic patients, has recently been shown to decrease both partner preference and alcohol preference in voles. These findings strongly suggest that mechanisms by which social factors influence drinking have biological roots, and can be studied using rapidly developing new animal models.

## 1. Introduction

Alcohol abuse is a serious and prevalent problem that warrants investigation into factors causing or maintaining related addictive behavior, in addition to factors that protect against excessive alcohol use, or that assist treatment of established drinking behavior. Social factors have crucial and long-lasting effects on alcohol intake which, in some instances, lead to excessive alcohol intake. For example, social stress of separation due to divorce or death of a loved one has been associated with increased alcohol intake [1–3]. On the other hand, a supportive social network is a major aide for abstinent alcoholics [4,5]. Under different circumstances, alcohol drinking is often increased in enjoyable social situations, and an individual’s social network and number of drinking buddies are related to his or her alcohol drinking level [6].

While it is difficult to dissociate the contribution of biological and cultural influences to the interactions between social conditions and alcohol intake in humans, a number of animal models have been developed that make it possible to not only observe the effects of specific social circumstances on alcohol intake, but also to evaluate the involvement of particular neural or genetic factors. Here we discuss findings from several animal models of social effects on voluntary alcohol intake which indicate that the social influences on alcohol drinking are not only cultural, and have given insight into how different types of social influences can affect alcohol drinking, and the biological mechanisms that may mediate these behaviors. Some of the social interactions addressed are negative, including separation from a mother, social isolation, crowded housing conditions, and social subordinance or defeat, while other interactions are positive, such as observation of a familiar cagemate, or affiliative sibling relationships. Both positive and negative social interactions have important effects on alcohol intake in humans and in the animal models discussed here.

## 2. Effects of Rearing or Early Life Stress on Alcohol Intake

The mother-infant bond is important for social development [7,8], and changes in this relationship can have long-lasting effects on the offspring [9]. Disruption of the mother-infant bond, through permanent or brief intermittent separation, leads to development of greater anxiety-like behavior in several species [10–13]. These species are useful models for the effects of early social stress on different aspects of behaviors, including alcohol intake.

### 2.1. Peer-Rearing in Primates

A number of studies have examined the effects of peer-rearing in rhesus macaques. These studies compared monkeys that were raised in the absence of the mother or other adults for the first six months of life, but had been housed in social groups of four peers, to monkeys that were raised by their mothers. Although peer-reared monkeys established bonds with their peers, they also exhibited more fear-related behaviors and decreased exploration compared to mother-reared monkeys. When the 50-month old animals were given one hour daily access to sweetened 7% alcohol four days a week, peer-reared monkeys drank significantly more than the mother-reared animals. However, when the animals were isolated, the mother-reared monkeys increased their alcohol intake to the level of peer-reared, which was not increased by the separation [14,15]. In addition to alcohol intake, peer-rearing affected a number of other behavioral phenotypes including aggression, impulsivity, and social behavior [16,17].

These studies indicate that early childhood social rearing experiences affect future predisposition to excessive alcohol intake. However, it should be noted that in this study, peer-reared monkeys had elevated plasma cortisol levels at baseline, and increased cortisol and ACTH concentrations during acute separations. Importantly, the peak cortisol levels during separation were positively correlated with alcohol intake [14]. Therefore, it is difficult to know whether the increased alcohol consumption in peer-reared animals was due specifically to the rearing conditions, or if it was a secondary effect, due to the elevated stress hormone levels. Non-social stressors, such as foot-shock exposure in adolescence, can affect alcohol intake in adulthood [18,19], indicating that different forms of stress may yield the same behavioral outcome. In the case of peer-rearing, this negative social environment mediates the stress-induced increase in alcohol intake. In this and other cases of negative social environments that will be presented in this review, it becomes difficult to determine what aspects of the social environment or stress response may be responsible for alterations in alcohol drinking.

### Serotonin

In addition to exhibiting high levels of alcohol intake, peer-reared non-human primates had lower CSF levels of serotonin metabolite 5-hydroxyindolacetic acid (5-HIAA) [15], which is associated with high levels of alcohol intake in humans and other primates [20]. Further, the selective serotonin reuptake inhibitor sertraline decreased alcohol consumption in peer-reared monkeys, while also disrupting the increased aggression observed in these animals [20]. These studies implicate a role for serotonin in mediating the behavioral effects of the peer-rearing experience.

The peer-rearing model described above has successfully elucidated a gene by environment interaction. Female macaques possessing the short allele for a polymorphism in the promoter region of the serotonin transporter (5-HTTLPR) exhibited a greater alcohol preference than those with the long allele, but only if they were peer-reared; mother-reared monkeys showed no effect of genotype on alcohol preference [21]. This indicates that the serotonin transporter plays an important role in modulating alcohol preference, since the genotype that results in lower mRNA levels of the gene corresponds to increased preference. Importantly, this finding also indicates that the effect of genotype can be buffered by the influence of mother-rearing.

Again, it is uncertain whether the effect is in fact due to the past social experiences, or to the elevated stress levels observed in peer-reared monkeys. Since the 5-HTTLPR is responsive to glucocorticoids levels, it is likely that this plays an important role in the gene by environment interaction detected. However, Barr *et al.* have also shown that animals with the short allele exhibit increased sensitivity to the ataxic and sedating effects of alcohol, independent of rearing condition [22], suggesting that the polymorphism may directly impact the effects experienced from alcohol, regardless of stress levels.

### 2.2. Early Weaning in Rats

Studies in rodents are inconsistent regarding the effects of early weaning or handling separation on later alcohol intake. For example, Rockman *et al.* showed an increase in alcohol intake in rats weaned early (at postnatal day 16) when tested for alcohol consumption in early adulthood, but only at higher doses (7 and 9% ethanol, but not 3 or 5%) [23], while Fahlke *et al.* showed a relative decrease in drinking in early-weaned rats compared to normally weaned rats at low concentrations (2, 4 and 6% ethanol, but not 8 or 10%), tested in adolescence[24]. The age at testing may play a role in the discrepancies observed, and the possibility of different effects on alcohol drinking at different concentrations should also be considered. In addition, there is evidence that a social stress such as early weaning may compound harmful effects of prenatal alcohol exposure [25].

## 3. Effects of Housing Conditions on Alcohol Intake

Independent of rearing conditions, social conditions during adolescence and adulthood affect alcohol intake. Studies in rats described below have revealed that isolation or crowding conditions, which both induce stress [26,27] can increase alcohol intake. Numerous studies examining the effects of isolation on alcohol intake have addressed not only the behavioral effects, but also the biological mechanisms that may mediate the interaction between this social stress and alcohol consumption. Namely, serotonin, GABA, and dopamine systems have all been implicated in affecting alcohol consumption in response to social isolation.

### 3.1. Isolation in Rodents

In a complex study of changes in housing conditions affecting alcohol intake, Wolffgramm and Heyne showed an increase in alcohol intake in isolated and contact-housed Wistar rats, compared to group-housed rats, and that housing changes resulting in increased levels of isolation (*i.e.*, grouping to contact caging or contact to isolation) corresponded with increased drinking [28]. Similarly, Hall *et al.* observed an increased consumption of 16% alcohol (but not lower concentrations) in adult Fawn–Hooded and Wistar rats isolated since weaning, compared to pair-housed controls, although there was no difference in alcohol preference due to the two housing conditions [29].

While a majority of reports on isolation in rats confirm the increased alcohol consumption following isolation described above [30–37], decades of literature examining effects of isolation on alcohol intake include some apparent contradictions; some studies report no difference in voluntary alcohol intake in response to isolation stress, while few even report a decrease. As suggested by Schenk *et al.*, there are a number of procedural differences that make direct comparisons between studies difficult. These authors were able to demonstrate the importance of age at isolation on subsequent alcohol intake, showing in Long-Evans rats that 12 weeks of isolation starting at weaning resulted in a considerable increase in alcohol intake compared to rats that had been housed four per cage for the 12 weeks preceding alcohol testing, while 12 weeks of isolation starting as adults did not affect alcohol intake [32].

More recent studies demonstrated the importance of the age at which social manipulations and testing are performed. Juvenile Wistar rats that had been continuously isolated exhibited a higher alcohol intake than rats that had been continuously socially-housed, socially-housed with intermittent isolation, or isolated with intermittent social contact, when tested during the same pre-pubescent period in which housing manipulations occurred. However, when the same animals were later tested in adulthood, the rats that had been socially-housed with intermittent isolation drank more than the other groups, but only when alcohol was available in social conditions. The remaining groups drank less in social conditions than in isolation, in adulthood [35]. This indicates that chronic isolation can induce higher drinking at a young age, while in adulthood drinking is increased in isolation regardless of previous social history, and social history can differentially affect drinking levels under social circumstances.

One contradictory study showed that alcohol intake was actually lower in isolated Wistar rats than pair-housed rats during forced consumption, while during subsequent two-bottle choice testing there was no difference [38]. In addition to the forced consumption, one distinction between this study and others is that the pair-housed animals were not only pair-housed preceding testing, but also throughout alcohol availability. Other studies have separated group-housed animals for testing of alcohol consumption. It is possible that the difference in social circumstances during testing can affect consumption, as demonstrated above [35]. However, the same lab tested alcohol preferring (P) and non-preferring (NP) rats in a similar manner and found that isolated preferring rats consumed more alcohol than paired preferring rats, while there was no difference in consumption between isolated and paired non-preferring rats [36], indicating that the mode of testing in pairs cannot be solely responsible for the discrepancy.

In contrast to the study described above by Ehlers *et al.* in selectively-bred alcohol preferring and non-preferring rats, Lodge and Lawrence reported that Fawn-Hooded rats that preferred alcohol did not differ in their alcohol consumption from isolated rats [39]. However, this could be due to a ceiling effect of alcohol consumption for this strain under these conditions, particularly since the control rats were divided into alcohol preferring and non-preferring subgroups based on their intake, and only the preferring rats were compared to isolated rats, which were not divided based on intake.

There are fewer studies examining the effects of isolation on alcohol intake in mice. Post-weaning isolation increased alcohol preference and intake in male C57BL/6J (C57) mice, tested as adults (60 days old) [40], and one week of isolation at 30 or 44 days of age increased alcohol consumption tested over the following two weeks in C57 and DBA/1 mice [41]. Together, these results indicate that isolation in adolescence or early adulthood can lead to increased alcohol intake in adult mice.

Many studies of the effects of isolation on alcohol intake have focused on the biological mechanisms contributing to the observed behavioral effects, and the serotonin, GABA, and dopamine systems, as well as HPA axis activation are each affected by the conditions of the social environment and play a role in the effect on alcohol drinking.

#### 3.1.1. Role of serotonin

Isolated alcohol-preferring Long-Evans rats exhibited a higher drinking level than rats that were group-housed, and the drinking level decreased when the animals were treated with clomipramine, an anti-depressant that inhibits uptake of serotonin [30]. In addition, there was more serotonin in the synaptosomes of alcohol preferring rats compared to non-preferring rats, and in those of isolated rats compared to grouped rats, indicating that not only may serotonin be important for innate alcohol reward, but also that it may be responsive to social stress, thus increasing drinking in response to isolation. More evidence for the effect of serotonin levels in response to social stress increasing drinking is presented in the context of social dominance interactions below.

A study examining the importance of serotonin in the comorbidity of depression and alcohol use assessed alcohol intake in old and young Sprague-Dawley rats exposed to four days of 1–4 hours of isolation in a novel environment. The 5HT_2A_ receptor antagonist nefazodone decreased isolation stress-induced alcohol drinking and returned elevated corticosterone levels to baseline [33]. Interestingly, in this study, alcohol intake and corticosterone levels remained elevated in the days of recovery following isolation stress in aged rats only, while young rats returned to baseline levels, indicating an important effect of age on chronic effects of stress, and further supporting the link between HPA axis activity and alcohol intake.

In C57 mice, Advani *et al.* recently showed that 5HT_1A_ receptor activity was upregulated in the dorsal raphe of adult male and female animals that had been isolated in adolescence, while only the isolated males had exhibited a consistent increase in alcohol preference and consumption, and isolated females in fact exhibited a decrease in alcohol consumption during the final two weeks of study [40]. This suggests that the serotonin system can display long-term alterations in response to isolation stress, but that the 5HT_1A_ receptor activity upregulation is not the only mechanism responsible for increased alcohol consumption, since the opposite effect on drinking was observed in female mice with similar receptor activity upregulation, compared to mice pair-housed in adolescence. In this study there was no effect of isolation on 5HT_1A_ receptor activity in the CA1, dentate gyrus, or median raphe, in part confirming results in rats by Rilke *et al.* in which no differences were discovered in binding or affinity of serotonin 5HT_1A_ receptors in the hippocampus, although they suggested that other areas could be affected [42].

#### 3.1.2. Role of GABA and modulatory steroids

Isolation results in a decrease in neuroactive steroids that can influence the structure and function of GABA_A_ receptors, and in turn affect the response to alcohol [43]. Alcohol increased cerebrocortical and plasma concentrations of neuroactive steroids to a significantly greater degree in Sprague-Dawley rats that had been isolated since weaning than group-housed rats, suggesting that the increased preference for alcohol in isolated rats may be due in part to the greater increase in neuroactive steroids such as progesterone metabolites that are known to be anxiolytic and may potentiate the central response to alcohol [44]. Studies of rats bred for alcohol preference or avoidance give further support for the role of neuroactive steroids in alcohol preference. Sardinian alcohol-preferring (sP) rats exhibited greater increases in levels of allopregnanolone and allotetrahydrodeoxycorticosterone in response to an acute injection of alcohol than Sardinian non-preferring (sNP) rats [45]. This supports the idea that the anxiolytic function of neuroactive steroids via GABA_A_ receptor activation contributes to alcohol preference.

The *in vitro* binding affinity of flunitrazepam was increased in the cortex of isolated and contact-housed Wistar rats compared to group-housed rats, indicating that the GABA_A_ receptor may be more potently activated by binding at the benzodiazepine site in isolated rats, although there were no differences in binding affinity as an effect of alcohol intake [42]. There is evidence that GABA_A_ receptor subunit composition changes following isolation [43], which could contribute to the differential binding affinity at the benzodiazepine site, while the altered concentrations of neuroactive steroids that may act as allosteric modulators mentioned above may also affect binding affinity [42]. In further support of a role for involvement of the GABA_A_ receptor benzodiazepine site in affecting alcohol consumption in isolated animals, Nuñez *et al.* showed that the anxiolytic alprazolam attenuated drinking in aged Sprague-Dawley rats that had increased alcohol intake following social isolation [34].

#### 3.1.3. Role of dopamine

In addition to the effects on GABA receptors, Rilke *et al.* showed alterations in dopamine D_2_ receptors in Wistar rats following isolation and/or alcohol intake. Short- or long-term isolation (one day or five weeks, respectively), contact housing (four adjacent wire cages), and group-housing with forced ethanol consumption (6% unsweetened ethanol) led to decreased B_max_ for the D_2_ receptors in the striatum, detected by [^3^H]spiperone binding, in comparison to group-housed rats. Interestingly, forced ethanol consumption in long-term isolated rats recovered B_max_ to the level of group-housed rats. These results suggest that isolation and alcohol may independently have the same effect, decreasing D_2_ receptor density, likely due to increased dopamine in the synapses, but that alcohol interferes with the further downregulation of receptors in chronically isolated animals [42].

#### 3.1.4. Interaction with the HPA axis

Social isolation in rodents is considered to be a negative stressful condition, but relatively few studies have examined the effect of isolation on the physiological stress response, namely the hypothalamic-pituitary-adrenal (HPA) axis. Basal levels of ACTH were lower in isolated male Sprague-Dawley rats, compared to group-housed animals, but the increase in corticosterone in response to administration of corticotropin-releasing factor (CRF) was significantly higher in isolated than group-housed rats, and the decrease in corticosterone in response to administration of dexamethasone, a synthetic glucocorticoid that normally exerts negative feedback on the HPA axis, was diminished in isolated rats compared to group-housed controls [46]. Together, these results indicate that basal HPA axis activity and response of the axis to a stressor may both be altered due to chronic isolation.

The study in Fawn-Hooded rats by Lodge and Lawrence [47] demonstrated an interaction between the isolation-induced dysregulation of the HPA axis and alcohol intake by showing that antalarmin, a CRF_1_ receptor antagonist, decreased alcohol preference and intake in isolation-reared animals. This finding indicated that the activation of the HPA axis via CRF_1_ receptors was important for the observed ethanol preference in isolated rats. Importantly, the anxiolytic diazepam, a GABA_A_ allosteric modulator, did not decrease the established alcohol preference or intake, confirming that the observed preference was not due to a general anxiety, but specifically to activation of the HPA axis.

In addition to effects of isolation on HPA axis functioning that can modulate alcohol intake, isolation can also mediate the effects of alcohol on the HPA axis. For example, isolated alcohol-preferring (AA) rats failed to show an increase in serum corticosterone levels in response to an injection of alcohol, while isolated alcohol non-preferring (ANA) rats did show an increase, and group-housed rats of both strains also exhibited an increase in corticosterone levels [48]. This implies that there may be complex interactions between the HPA axis and alcohol intake that are influenced by social isolation.

### 3.2. Crowding in Rodents

While the social stress of isolation can lead to increased alcohol intake, studies have shown that social stress due to crowding can also increase drinking in rodents. Water-deprived female Sprague–Dawley rats housed eight per cage and given individual 10 minute access to 10% alcohol and water twice daily drank a higher dose of alcohol during the last half of an 18-day experiment than rats individually housed and exposed to the same conditions of water deprivation and alcohol exposure [49]. In a different paradigm, male Sprague–Dawley rats that were moved from individual housing to pair housing without an increase in cage size escalated their intake of continuously-available 10% alcohol from a stable baseline established in isolation. Notably, neither administration of ACTH nor of synthetic glucocorticoid dexamethasone induced an increase in individually-housed rats (or pair-housed rats), suggesting that the increase in alcohol intake observed in paired rats was not due to ACTH or the subsequent increase in glucocorticoid levels alone and that the stress response to increased crowding or social interaction is not solely responsible for the increased alcohol consumption [50].

Despite the probable independence of effects of pair housing on alcohol drinking observed in this study, it is difficult to distinguish these from potential effects of novelty and environmental enrichment. Thus, in addition to isolation or crowding, studies with Maudsley Reactive [51] and Sprague–Dawley [31] rats have shown an increase in alcohol intake in seminatural or enriched housing conditions, in which rats are housed with many other animals, but with plenty of room and other stimuli available as well, even above intake levels of isolated rats.

## 4. Effects of Social Dominance Interactions on Alcohol Intake

While the presence or absence of certain social relationships impact alcohol intake as described above, the nature of social interactions is also important. Numerous studies have examined the effects of social dominance or subordinance on alcohol intake. Dominant or subordinate roles in rodents are typically established and/or assessed during aggressive contact episodes. Some studies have observed natural behavior in previously established groups, while others have used forced interactions between animals not previously housed directly together, with some disparate results.

### 4.1. Role of Dominance in Established Colonies

While most studies of social dominance relationships have focused on rodents, primates also exhibit stable dominance hierarchies and can be useful for examining the effects of rank on alcohol intake. One study of social rank in squirrel monkeys living in colonies of 4–10 individuals has revealed that there is a negative correlation between the index of dominance and the amount of alcohol consumed [52]. The relatively higher consumption of alcohol in subordinates is consistent with effects observed in rodent species. In primates and rodents, there is evidence that subordination is linked with heightened HPA axis activity [53,54], further supporting the hypothesis that increased stress leads to increased alcohol intake.

One study examining social behavior in Long-Evans rats in established colonies found that in each of 10 colonies containing five males and three females, one male displayed a greater degree of aggressive behavior relative to defensive behavior in interactions with other males, and was considered dominant. These dominant rats consumed significantly less alcohol than subordinate rats did at both concentrations tested (4% and 8%) [55]. One interpretation the authors provide is that the difference in anxiety levels of dominant and subordinate rats affects alcohol intake, such that the more anxious subordinate rats consume more alcohol, to act as an anxiolytic, than dominant rats, which perceive less reward from alcohol intake. Notably, females in these colonies drank significantly more than males. This finding is in agreement with many studies in mice and rats showing that females consume more alcohol than males, and it also fits the proposed theory relating stress and anxiety to alcohol intake, since females are not the dominant animals in the colony. This group subsequently showed that levels of serotonin metabolite 5-HIAA were elevated in limbic brain regions and the spinal cord of subordinate rats compared to dominant or control (isolated) rats, indicating that increased serotonin turnover is linked with increased consumption [56], similar to findings in isolated rats described above. The negative correlation between dominance rank and alcohol intake in rats has been confirmed by others, in Wistar rats [28].

In male C57 mice exposed to seven consecutive days of confrontations by different pairings of dominant and subordinate animals, subordinate mice consumed more 20% alcohol than aggressive mice, particularly in the second of two weeks of alcohol testing, while CBA/Lac mice, a low-preferring strain, showed no effect of social role or experience on alcohol intake [57]. These results are repeatable for C57s [58] and are consistent with findings in rats, but they also show that the effect of social defeat or subordinance may not universally increase alcohol intake, since low-preferring animals did not alter their intake.

### 4.2. Role of Social Defeat

Several studies report contradictory results of subordinance on alcohol intake; however, the differences can be at least partially explained by methodological differences and consequent differences in what is labeled ‘subordinate’ behavior. The studies in rodents described above refer to dominance and subordinance in the context of colonies of peers, where stable roles exist but are not imposed, or in the context of dyads of peers, where relative behaviors are examined. In contrast, other studies have sought to explore the effects of imposed subordinance on alcohol intake with variations of a resident-intruder procedure, where the subject is the intruder in the home cage of an older, larger rat that has been selected for aggressive behavior. In these studies, the authors report a decrease in alcohol intake in subordinate rats [59,60]. It is possible that alcohol drinking following social defeat is affected differently than alcohol drinking in naturally established subordinates, explaining the discrepancy.

However, one problem still remains. The presumption is that subordinance in a colony resulting from social interactions and individual variability, induced subordinance resulting from resident-intruder attacks, or even social isolation and maternal separation, are all social stressors that lead to elevated stress responding and anxiety levels, which animals attempt to alleviate with increased alcohol consumption. The studies involving social defeat show the opposite effect, where defeated animals attenuate their alcohol intake, in spite of reports that this procedure does have significant acute [61] and lasting effects on HPA axis activity [62,63], and increases anxiety-like behaviors [64,65], contradicting the presumption that alcohol intake is increased as a way to cope with anxiety or tension resulting from stress.

In light of this contradiction, it stands to reason that different types of social interactions can affect alcohol drinking without direct involvement from the HPA axis. However, as we have noted, the social manipulations discussed thus far are all associated with stress, including dysregulation of the HPA axis and anxiety-related behaviors. Therefore, it is difficult to assess the effects of the specific social interaction with certainty using these models.

## 5. Social Facilitation of Alcohol Intake

Although studies described above suggest that the effects of social factors on alcohol intake have biological underpinnings, all of the described experimental manipulations (early separation from a mother, crowding, social isolation, and social defeat) have negative connotations and are accompanied by increased anxiety and stress. Therefore, they do not allow distinction between a direct effect on alcohol intake and stress-mediated effects. However, in the human experience, there are many enjoyable social situations which often lead to alcohol intake and in some cases to excess drinking. Moreover, modeling such positive social situations in rodents could allow distinction of the effects of social factors from stress.

### 5.1. Demonstrator-Observer Rat Models of Alcohol Acceptance

Rats will exhibit a greater preference for a novel substance when they are allowed to observe another rat that has been exposed to the substance [66–68]. This demonstrator-observer paradigm has been extended to assess effects related to alcohol preference, but interestingly, alcohol odor preference was only increased in adolescent rats that had been able to interact with an alcohol-intoxicated peer, not in those that were exposed to an anesthetized rat that had also received alcohol [69]. Furthermore, alcohol preference was increased in adolescent male Sprague-Dawley rats that had been allowed to observe and interact with an intoxicated familiar cagemate, whereas alcohol preference was decreased in rats that had observed and interacted with an intoxicated unfamiliar peer [70]. This indicates that the relationship with the demonstrator is an important factor in interpretation of the stimulus substance. Interestingly, the relationship was not important for female adolescent rats, which exhibited an increased preference for alcohol after exposure to either a familiar cagemate or an unfamiliar peer. While in some cases familiarity may be important, these demonstrator-observer studies performed in rats have the disadvantages of, first, eliminating the possibility of interactions during drinking that may affect alcohol intake, and second, not allowing study of the effects of specific social affiliations on alcohol drinking.

### 5.2. Rodent Models of Specific Social Affiliations

One of the drawbacks of modeling social affiliation in traditional laboratory animals is that most rodents do not form affiliations with specific individuals. While mice and rats do prefer environments associated with social context during adolescence [71–73] and in adulthood during sexual interactions [74,75], mother-infant bonding [76] and even aggression [77], and can show signs of anxiety- and depression-like symptoms when they are socially isolated [78], there is no evidence that they show strong pair bonds with, or prefer to spend time with, a particular individual. However, in recent decades, specific bond formation has been studied in another rodent genus, the vole.

## 6. Vole Models of Affiliative Relationships and Alcohol Intake

Some species of the genus Microtus (voles), including prairie (*M. ochrogaster*) and pine voles (*M. pinetorum*), show remarkable pair bonding behavior. Most specifically, mating in these species leads to a life-long formation of a breeding pair that shares the same nest and territory where they are in frequent contact. Males of these species participate in parental care, and intruders of either sex are rejected [79–81]. This social bond is not common to most rodent species; for instance, mating among laboratory mice, rats, and other microtine rodent species, such as meadow (*M. pennsylvanicus*) and montane voles (*M. montanus*), does not induce pair bond formation [82,83]. Therefore, prairie and pine voles have an advantage over most other rodent species in that they clearly form specific affiliations.

### 6.1. Overlap in the Neurobiology of Affiliation and Drug Reward

The partner preference test has been developed to assess the strength of a pair bond in voles in the laboratory. This test compares the time the animal spends with a partner versus time spent with a stranger after a period of cohabitation with the partner [84]. Studies using this test have examined the effects of pharmacological manipulations on pair bond formation to elucidate a variety of neurobiological substrates involved in the social bond, which have been recently reviewed [85]. Importantly, many of these molecules are known to play a role in addiction as well. For example, the neuropeptide arginine vasopressin acting on central V1a receptors facilitates pair bond formation in male prairie voles [86], and is implicated in addiction, particularly alcohol intake [87]. Alcohol directly affects the release of vasopressin [88], and vasopressin is thought to play a part in modulating alcohol preference [89] and tolerance [90,91]. Oxytocin facilitates partner preference formation in female prairie voles [92], and has a number of addiction-related effects similar to vasopressin, including modulation of alcohol tolerance and dependence [93]. Together with oxytocin, dopamine is required for pair bond formation [94–96]. Activation of D1 receptors in the nucleus accumbens blocks partner preference formation, while activation of D2 receptors facilitates this formation. A large number of studies have demonstrated that the dopamine system is one of the neurotransmitter systems critically regulating addiction to various drugs [97–101], including alcohol [102]. The CRF system, a system regulating the HPA axis, is also implicated in regulation of pair bond formation. Importantly, central administration of CRF, in doses not affecting anxiety levels, promotes pair bond formation, while central administration of CRF antagonists attenuates these effects [103,104]. It is also well known that components of the CRF system regulate alcohol intake [105,106].

Preliminary evidence has shown that the opioid system may also be involved in pair bond formation despite relative resistance to manipulations of the opioid system in prairie voles [107]. Thus, female prairie voles injected with 7.5 mg/kg naltrexone, an opioid receptor antagonist, three times throughout the 18-hour cohabitation period did not exhibit a partner preference in the test following cohabitation, and exhibited a preference for the stranger, while the majority of vehicle-treated voles displayed a partner preference [108]. These recent studies are significant because the opioid system is important for regulation of alcohol-associated reward, and naltrexone is the first centrally-acting treatment approved for alcoholism.

### 6.2. Prairie Voles as a Model to Study Drug and Alcohol Addiction

Given the substantial overlap between mechanisms involved in formation of social bonds and addiction, the prairie vole appears to be a useful model for examining the biological mechanisms underlying the effects of social relationships and social stress on addiction-related behaviors.

Recent studies have begun to examine drug reward in prairie voles. One study demonstrated that male and female prairie voles exhibited a conditioned place preference for a cage floor that had been paired with amphetamine [109]. Another study from the same laboratory showed that amphetamine did not induce a partner preference unless a dopamine D1 receptor antagonist was pre-administered, and also showed that prairie voles had a more robust, long-lasting response to amphetamine, indicated by an increase in dopamine release in the nucleus accumbens, than non-monogamous meadow voles [110]. These results indicate that since social reward and drug reward utilize some of the same pathways, animals that have more developed natural (social) reward systems may be more sensitive to drug reward.

Our laboratory has demonstrated that pair-housed prairie voles exhibit a high preference for alcohol, and that they consume similar doses of alcohol to C57BL/6J mice, a mouse strain known for their high alcohol consumption [111]. Since naltrexone is used as a treatment for human alcoholics and has been shown to disrupt pair-bond formation in a study described above, recently we sought to test the effects of this drug on alcohol intake in prairie voles. First, prairie vole siblings were housed together for five days with continuous access to water in 25 mL glass tubes to acclimate them to the drinking tubes before testing. Then the pairs were moved to new cages where they were housed together, separated by a wire mesh through which they could interact, but which would allow monitoring of each individual’s drinking. For the following four days, each vole was given two-hour access to two bottles, one containing tap water and another containing 10% (volume/volume) ethanol in tap water at the onset of the light cycle, and continuous access to one tube containing water for the remaining 22 hours per day. The positions of the water and ethanol tubes were switched daily to avoid a side preference. Fluid levels were recorded at the start and end of each two-hour drinking session each day, and the volumes consumed were used to calculate the alcohol preference ratio and dose consumed. Each day, intraperitoneal injections were given 20 minutes prior to the drinking session. On the first two days, saline injections were given, and on the third and fourth days half the voles received naltrexone (8 mg/kg) while the other half received saline. Siblings housed together received the same drug treatment.

**Figure 1.**
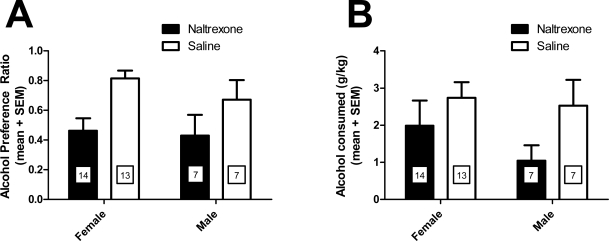
**(a)** Alcohol preference ratio and **(b)** alcohol dose consumed by female and male prairie voles administered saline or naltrexone (8 mg/kg) in a two-hour limited access procedure. The number of animals per group is noted within each bar.

Voles exhibited a clear alcohol preference, similar to C57BL/6J mice, and in contrast to a majority of other rat and mouse strains. Moreover, naltrexone significantly decreased alcohol preference [F(1,37) = 9.27; p < 0.005], and there was a trend toward a decrease in the alcohol dose consumed [F(1,38) = 2.954; p = 0.0938] (Figure 1), indicating that the opioid receptor antagonist decreases alcohol drinking as it does in humans. These results are intriguing as they suggest that pharmacotherapies approved in human alcoholics also decrease alcohol drinking in prairie voles, a species know to form specific pair-bonds. Therefore, the prairie vole may be very useful for understanding the biological roots of social aspects of alcohol consumption.

## 7. Conclusions

Animal models of social effects on alcohol intake have given us valuable evidence that these effects are influenced by a variety of biological mediators, independent of cultural influences that may also affect alcohol intake in humans. Serotonin, dopamine, and GABA systems play roles in mediating the effects of social separations or social rank on alcohol intake. This literature is not without contradictions, but some general conclusions can be drawn. Most studies involving social separation, either by early separation from a mother or by complete isolation, demonstrated elevated alcohol preference or intake. Some of the contradictory results may be explained by procedural differences, including the age at which isolation and testing occur. Subordinance has also been linked with elevated alcohol intake, but social defeat leads to suppression of alcohol drinking.

While these models have shown the importance of biological factors in mediating the effects of social circumstances on alcohol intake, most of them fail to allow a certain distinction between the effects of social factors and the general stress response that is induced in each of the models. Newly developed animal models that can assess social influences of drinking under non-stressful conditions will be invaluable for elucidating the biological underpinnings of social effects on alcohol intake. By utilizing the strong social bonds exhibited in prairie voles, the effects of specific affiliative relationships on drinking behavior can be assessed, which is important since social relationships in humans can play critical roles in excessive drinking. With the ability to model these important social factors, the understanding of the mechanisms involved in their effects on alcohol intake will continue to grow.
